# Fatal Dengue Myocarditis despite the Use of Extracorporeal Membrane Oxygenation

**DOI:** 10.1155/2016/5627217

**Published:** 2016-11-28

**Authors:** Yee-Huang Ku, Wen-Liang Yu

**Affiliations:** ^1^Department of Internal Medicine, Chi Mei Medical Center, Liouying, Tainan, Taiwan; ^2^Department of Intensive Care Medicine, Chi Mei Medical Center, Tainan, Taiwan; ^3^Department of Medicine, School of Medicine, College of Medicine, Taipei Medical University, Taipei, Taiwan

## Abstract

Dengue is an important mosquitoes-borne viral disease which is endemic in tropics and subtropics region. Rapid spreading of disease to previously unaffected region was found in recent years. Atypical manifestations, such as myocarditis, were reported during large outbreak. There is a wide range of clinical manifestations of cardiac involvement in dengue, but rarely fatal. Here we reported a case of fulminant dengue myocarditis in fatal outcome despite cardiac mechanical support.

## 1. Introduction

Clinical manifestation of dengue ranges from self-limited fever to severe hemorrhagic manifestations and even shock syndrome. Cardiac involvement in the patients with dengue fever is not uncommon, but fatal outcome is rare. There is also limited report on using cardiac mechanical support. Here we reported a case of fatal dengue myocarditis despite cardiac mechanical support.

## 2. Case Presentation

A 52-year-old woman was admitted to our emergency department (ED) on day 3 of chest pain. She had febrile episode and sore throat 5 days ago, followed by the development of chest discomfort, diaphoresis, nausea, and vomiting. The patient denied recent travel history. She had received operation for breast cancer and completed chemotherapy 10 years ago and was considered healthy otherwise.

On arrival at our ED, vital signs were body temperature, 36.6°C; pulse rate, 90 beats per minute; respiratory rate, 26 breaths per minute; and blood pressure, 103/66 mmHg. Laboratory data revealed white blood count cell, 3100/*μ*L with band 1%, segment 6%, and atypical lymphocyte 2%; hemoglobin level, 15.6 g/dL; hematocrit, 46.4%; platelet count, 116000/*μ*L; creatinine, 0.9 mg/dL; alanine aminotransferase, 32 IU/L; troponin-I, 1.29 ng/mL (normal range < 0.05 ng/mL); and c-reactive protein, 1.7 mg/L. CXR did not show active lung lesions. Chest computed tomography scan excluded aortic dissection. Troponin-I level was rising to 4 ng/mL, so, as the ST-T changes in electrocardiogram (EKG) series, the patient was admitted to our intensive care unit.

While in intensive care, her hemodynamic status was unstable and required fluid resuscitation. Follow-up troponin-I was 6.5 ng/mL, series of EKG changes indicated acute myocardial infarction and acute pericarditis, and echocardiogram showed anteroseptal left ventricle hypokinesia with an ejection fraction (EF) of 34%. Cardioangiography was performed but revealed patent coronary arteries. However, the patient was in shock status. Arterial blood gas analysis showed severe metabolic acidosis (pH 6.917, base excess −27.2) and lactate was 18.6 mmol/L. She received endotracheal intubation for respiratory support. Intra-aortic balloon pumping (IABP) and extracorporeal membrane oxygenation (ECMO) were implanted for hemodynamic support as poor response of 3 vasopressors. Initial blood cultures revealed negative results. Serum dengue virus-real-time PCR (The Applied Biosystems® StepOnePlus™) showed positive in the second day. Other titers of* Mycoplasma pneumoniae*,* Chlamydia*, and* Legionella* and viral culture of throat swab and even sputum aerobic pathogen survey were negative. Her heart function deteriorated rapidly to an EF of 10% and severe pulmonary edema occurred. Dengue myocarditis was highly suspected. Intravenous immunoglobulin was administered. However, the patient finally died in the sixth day.

## 3. Discussion

Several dengue infection outbreaks have recently been reported in Taiwan, especially at southern Taiwan [1]. Overall, dengue fever is easy to diagnose when the patients are presenting with classical symptoms. However, on occasion, some patients present with atypical manifestations especially in a large number of cases occurring [1]. The patient we reported here presented with an atypical symptom which was mimicking an episode of acute coronary syndrome.

Clinical manifestations of dengue myocardial involvement range from mild elevation of biomarkers to myocarditis and even pericarditis [2]. ST-T segment change in our patient's EKG along with regional abnormal wall motion, mimicking acute myocarditis, is very rare [3]. Although ECMO has been reported to be beneficial in myocarditis-related cardiac arrest [4], there is limited report on dengue myocarditis with cardiac mechanical support as concerning fatal bleeding tendency of dengue-associated thrombocytopenia [5]. As there were no bleeding events, both IABP and ECMO devices were implanted, but our patient could not survive because of multiorgan failure.

In conclusion, new treatment strategy for fulminant dengue myocarditis is needed in the future especially in the era of increasing spread of dengue disease.

## References

[1] Chen C. M., Chan K. S., Yu W. L. (2016). The outcomes of patients with severe dengue admitted to intensive care units. *Medicine*.

[2] Miranda C. H., Borges M. D. C., Matsuno A. K. (2013). Evaluation of cardiac involvement during dengue viral infection. *Clinical Infectious Diseases*.

[3] Gulati S., Maheshwari A. (2007). Atypical manifestations of dengue. *Tropical Medicine & International Health*.

[4] Cheng R., Hachamovitch R., Kittleson M. (2014). Clinical outcomes in fulminant myocarditis requiring extracorporeal membrane oxygenation: a weighted meta-analysis of 170 patients. *Journal of Cardiac Failure*.

[5] Lin T.-C., Lee H.-C., Lee W.-H., Su H.-M., Lin T.-H., Hsu P.-C. (2015). Fulminant dengue myocarditis complicated with profound shock and fatal outcome under intra-aortic balloon pumping support. *The American Journal of Emergency Medicine*.

